# Georgia Lunatic Asylum

**Published:** 1872-03

**Authors:** 


### GEORGIA LUNATIC ASYLUM.

We are informed that Drs. James F. Bozeman and William Henry Cumming have been appointed to examine into and report upon the management and condition of the State Lunatic Asylum. From the damaging impressions which have existed in regard to this institution for years, thus unsettling the confidence of the profession and the people, it is eminently fit and proper that this investigation should be made; indeed, it is imperatively due, not only to the unfortunate class for whose proper care it was provided, but to the entire people of the State and the officers to whose hands so sacred a trust has been committed.

In the selection of a committee requiring such rare qualifications, the Governor of the State has exhibited a marked judgment and discrimination. We know but few gentlemen in the profession who combine to so great an extent the professional and financial knowledge with the sterling integrity required for the discharge of so delicate and important a duty. We shall be certain of getting the *facts*, which will either reëstablish the institution in the confidence of the people and the profession, or confirm the impressions which, if correct, make it a discredit to the State.

